# An Estimation of the Antiviral Activity and Toxicity of Biologically Active Substances Obtained from the Raw Materials of *Artemisia cina* Berg. In Vitro and In Vivo

**DOI:** 10.3390/molecules28145413

**Published:** 2023-07-14

**Authors:** Murat Zhurinov, Dmitriy Berillo, Kulzada Begalinovna Bazhykova, Kayrolla Dyusenbaevich Rakhimov, Tolkyn Bekezhanova

**Affiliations:** 1 D.V. Sokolsky Institute of Fuel, Catalysis and Electrochemistry, JSC, Almaty 050010, Kazakhstan; 2 Department of Chemistry and Biochemical Engineering, Institute of Chemical and Biological Technologies (IHBT), Satbayev University, Almaty 050013, Kazakhstan; 3 Department of Chemistry and Technology of Organic Substances, Natural Compounds and Polymers, Al-Farabi Kazakh National University, Almaty 050000, Kazakhstan; 4 Department of Engineering Disciplines and Good Practices, Asfendiyarov Kazakh National Medical University, Almaty 050000, Kazakhstan

**Keywords:** *Artemisia cina* Berg., biologically active substances, SARS-CoV-2, antiviral activity, acute toxicity

## Abstract

Species of the genus Artemisia are well known for their use as ingredients in ancient medicine. The advantage of using plant extracts compared to individual pharmaceutical ingredients is the rate of adaptation of the pathogenic microorganisms to the drug. Due to the rapid development of multidrug-resistance in microorganisms in the field, it is essential to search for novel, effective drugs with low toxicity. Therefore, the purpose of this study was to isolate and study the biologically active substances obtained from various substances in the raw materials of *Artemisia cina* Berg. The identification of the main biologically active components was performed using the method of chromato-mass spectrometry. Moreover, the antiviral activity of several extracts was studied using the method of measuring limiting dilutions (the Reed–Mench method), with some modifications. For the first time, the biological activity of extracts from the raw material of *Artemisia cina* Berg. upon the SARS-CoV-2 virus was confirmed. All the obtained extracts exhibited nontoxic effects in animals, with an LD_50_ greater than 2 g/kg. Comprehensive toxicological analyses are also presented in the study, such as those of the biochemical parameters of urine after one day and one week of the extracts’ administration in mice at a dose of 2 g/kg body weight. In all groups of animals that received extracts of *Artemisia cina* Berg., a slight increase in the presence of red blood cells in their urine was observed one day following the administration of the extracts. This increase decreased somewhat after a week; however, it remained higher than the levels observed in the control animals. In the three groups, there was also a slight increase in the amount of ketones in the urine. Two weeks following the administration of the extracts to these groups, the internal organs of the animals were examined. The examination showed that the internal organs of the animals that received the extracts were not visibly different from those of the control animals in terms of their size or appearance. The weight of the internal organs of the animals that received the extracts was also similar to the weight of the internal organs of the control animals, illustrating the absence of toxicity.

## 1. Introduction

Despite the success of the global vaccination of the population, the significant number of people who have already been exposed to the virus in the past, and the growth of herd immunity, new cases of SARS-CoV-2 continue to be registered in the data. Considerable efforts made by the scientific community have been directed toward obtaining antiviral drugs that are active against SARS-CoV-2; however, these efforts have been only marginally successful. The SARS-CoV-2 (COVID-19) global pandemic has killed millions of people and evolved numerous pathogenic strains. A recent comprehensive review stated that 23 Artemisia species have been described as possessing antiviral activity against seventeen different types of viral diseases. A total of 17 out of 23 antiviral Artemisia species were included in the ITS phylogeny. Ten antiviral Artemisia species appeared within the subgenus Artemisia clade; two species appeared within the subgenus *Absinthium clade*; three species appeared within the subgenus Dracunculus clade; and two species appeared within the subgenus *Seriphidium clade* [1].

*Artemisia annua* L. revealed some promising efficiency effects for the treatment of SARS-CoV-2, due to artemisinin showing a significant inhibition of 3C-like protease activity, but not Spike/ACE-2 binding. Moreover, the immunosuppressive effects of artemisinin on the TNF-α production in both pseudoviruses and lipopolysaccharide (LPS)-induced THP-1 cells were observed [2]. A hot water extract of *Artemisia annua* L. *cultivars* contains artemisinin in the range of 1.4–25.0 μM, and was effective against all five SARS-CoV-2 variants. The IC50 and IC90 values based on dried leaf weight utilized to produce the tea infusions were within the ranges of 11.0 to 67.7 μg and 59.5–160.6 μg of dry weight, respectively [3]. The same research group has reported that hot water leaf extracts based on artemisinin, flavonoids, or dry leaf mass have antiviral activity IC50 values of 0.1–8.7 μM, 0.01–0.14 μg, and 23.4–57.4 μg, respectively. Nevertheless, it was also noticed in the research that antiviral activity did not correlate with the artemisinin or total flavonoid contents of the extracts [4]. *Artemisia annua* L. can contain artemisinin in the range of 5825 to 7972 mg/kg. It is important to efficiently remove impurities (such as chlorophyll, etc.) to keep the valuable product at a high yield [5]. *Artemisia capillaris* (AC) extract was tested for its antiviral activity against the dengue virus, Zika virus, and Japanese encephalitis virus (JEV). It was established that *Artemisia capillaris* has inhibitory activity against JEV, ZIKV, and DENV replications. Moreover, the extract induced a reduction in viral RNA levels in a dose-responsive manner, with noncytotoxic concentrations ranging from 0.1 to 10 mg/mL, and possessed low cytotoxicity to Vero cells, with CC_50_ values of 33 ± 1.6 and 30 ± 1.7 mg/mL, respectively [6].

*Artemisia afra*, *Artemisia absinthium*, and *Pittiosporum viridflorum* leaves were investigated for their antiviral, antioxidant, and antipyretic activity. *Artemisia absinthium* extract exhibited the best activity against the A/Sydney/5/97 strain, while *Artemisia afra* extract illustrated the highest antioxidant potential against the tested antioxidant parameters. It has been reported that crude extracts significantly reversed yeast-induced pyrexia in rats, comparable to the model drug [7].

To date, acyclovir is the main clinical drug used in the treatment of Herpes simplex virus (HSV) infections. The failure of therapy in immunocompromized patients is caused by ACV-resistant HSV-1 strains, and there is a need for the development of novel anti-HSV-1 drugs. *Artemisia argyi* is historically known for its anti-inflammatory activity, as well as its antimicrobial and anticancer biological activity. Recently, it was illustrated that 10 μg/mL leaf extract exhibited potent antiviral effects on both normal and acyclovir-resistant HSV-1 strains [8]. *Artemisia cina* extract showed the most promising anti-H5N1 activity, with a highly safe half-maximal cytotoxic concentration of 50% (10 mg/mL) and an IC50 of 3.42 μg/mL. Additionally, the in vitro screening evaluation against influenza A/H5N1 illustrated an IC50 of 1.701 μg/mL, and an IC50 of 2.91 μg/mL for influenza A/H1N1 [9,10]. *Artemisia campestris subsp. glutinosa* (*Besser*) displayed anti-HIV activity in vitro (IC50 14.62 μg/mL). Researchers isolated two terpenes, damsin and canrenone, and four flavonoids: 6, 2′, 4′–trimethoxyflavone, acerosin, cardamonin, and xanthomicrol [11]. It was observed that a 50% aqueous methanol extract of *Artemisia rupestris* L. contained forty-four compounds, comprising eight caffeoylquinic acid derivatives, thirteen flavonoids, fifteen monomeric and dimeric sesquiterpenoids, four fatty acids, and two penylpropanoids [12]. One of the pieces of evidence for the recognition of the value of plant sources is the awarding of the Nobel Prize in Medicine and Physiology for the discovery of artemisinin, a drug that has an effect on the causative agent of malaria, obtained from *Artemisia* [3].

In this study, the in vivo potential of different herbal extracts of *Artemisia cina* Berg. has been evaluated, including the suppression of SARS-CoV-2 virus replication.

## 2. Results and Discussion

Phenolic compounds have also been discussed in the literature to possess an antiviral potential against viruses that affect the respiratory system. The recent review discusses the data concerning various plant extracts (*Angelica keiskei*, *Broussonetia papyrifera*, *Salvia miltiorrhiza Bunge*, *Bupleurum falcatum* L., etc.) of IC50 used against the SARS-CoV-2 strain; moreover, phlorotannins isolated from the edible brown algae *Ecklonia cava Kjellman* illustrated the same activity [13]. Regarding the production of extracts obtained from the raw materials of wormwood, using the results of the previous study [14,15], we developed a universal technology for obtaining thick extracts from the studied plants, and then a technology for obtaining dry extracts using a freeze-dryer. The advantage of the developed technology was the use of the ultrasonic extraction method, instead of the well-known maceration method. Therefore, we observed that ultrasonic extraction significantly reduced the extraction time and diminished the consumption of reagents. In addition, the developed technology made it possible to obtain dry extracts. The main advantage of dry extracts (compared to thick extracts containing a high level of moisture) was the ability to accurately calculate the concentration of a substance that was crucial for studying antiviral activity, as well as sample preparation for the following analytical evaluation, performed using GC–MS (Figures S5–S7). The schemes for *Artemisia cina* Berg. Extract preparation are presented (Figures S1–S4). The TLC analysis of *Artemisia cina* Berg. Extract is summarized in Table S1.

The qualitative and quantitative analyses of the obtained *Artemisia cina* Berg. Extracts were investigated using spectrometric and chromatographic analyses. Thin-layer chromatography (in comparison to standard compounds) confirmed the following classes of compounds were observed in the raw materials of *Artemisia cina* Berg: sesquiterpene lactones, terpenoids, and flavonoids. GC–MS was applied to analyze the qualitative and quantitative compositions of the following extracts obtained from the wormwood: No. 3, 4, and 7 (Table 1, Table 2 and Table 3).

The main component of extract No. 3 was estimated as α-santonin (66.33%), with a retention time of 18.2 min (Table 1). The extract also contained a relatively small amount of the compounds 2,3-Dihydro-4H-pyran-4-one (3.2%) and 3,5,5-trimethylcyclohexylisophosphofloride (6.73%) (which were not previously observed in plants), as well as lumisantonine (3.74%), and 6-nitro-2-phenyl-4-quinolinol (8.0%). Previously, 6-nitro-2-phenyl-4-quinolinol was reported to be observed in the research [15]. 2,3-Dihydro-4H-pyran-4-one derivative was previously observed in the leaves of *Bryophyllum pinnatum* [16]. *Lumisantonin* was previously detected in *Artemisia sericea*, *Artemisia tenuisecta*, and *Artemisia ifranensis* [17].

It was observed that extract No. 4 contained α-santonin (39.39%), butanoic acid (10.05%), and anhydro-β-retinol (3.28%) (Table 2).

The main components of extract No. 7 were α-santonin (66.35%), 4-H-pyran-4-one-2.3 (4.9%), 7-ethyl-4-nonanone (5.9%), and lumisantonine (2.58%) (Table 4). A comparative analysis of the qualitative and quantitative compositions of wormwood extracts indicated that all substances contained α-santonin in their composition, while its content in extract No. 4 was two times less than in extracts No. 3 and 7, respectively. Moreover, the common components of extracts No. 3 and 7 were 4-H-pyran-4-one-2,3 and lumisantonine. Taking into account the fact that, according to antiviral activity, the extracts were arranged in a row (No. 4 > No. 3 = No. 7), it can be assumed that their active ingredients were associated with the presence of santonin.

### 2.1. Evaluation of Biological Activity against SARS-CoV-2

**Figure 1:**
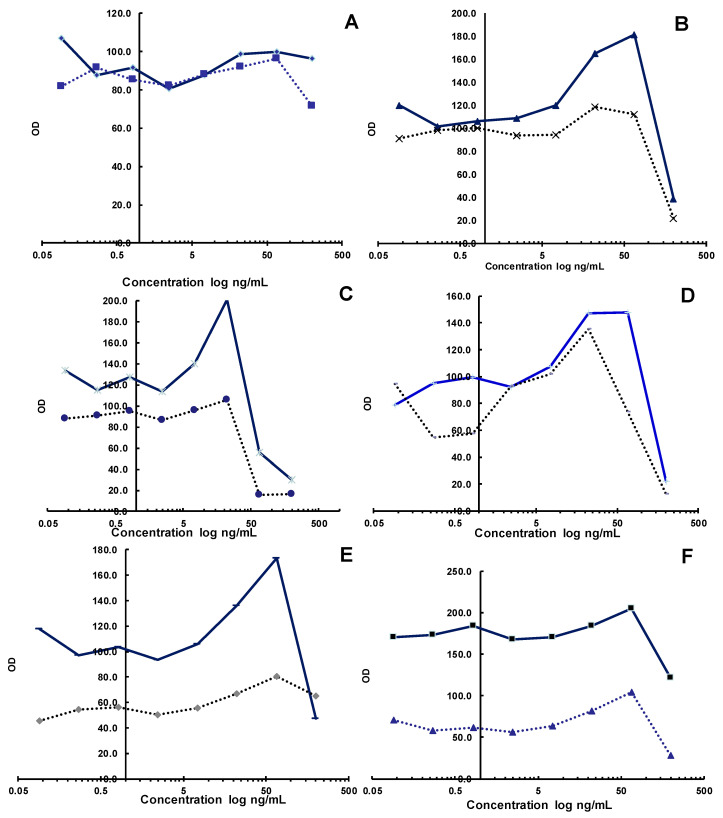
Plot of mean OD in wells of rows A–H versus mean OD of infected control wells (row 11). On the x-axis are the rows of the tablet (from left to right, and in the direction of increasing the concentration of the substance). On the y-axis is charted the ratio of optical densities. Dilutions of the Artemisia extract: (**A**) N2; (**B**) N3; (**C**) N4; (**D**) N5; (**E**) N6; (**F**) N7.

The substances obtained from aqueous extract No. 3 had an antiviral effect at concentrations of 0.37–3.33 mg/mL; however, at a concentration of 1%, the extract was toxic. The extract N3 illustrated IC_50_ at approximately 200 μg/mL. The hot water extract of *Artemisia cina* Berg. No. 4 exhibited an antiviral effect at concentrations of 0.12–1.1 mg/mL and an IC_50_ value equal to 75 μg/mL. An antiviral effect was pronounced, and it was concentration-dependent in this concentration range. However, the concentration ranges of 3.3–10 mg/mL for substance No. 4 were toxic to cells. Extract No. 6 revealed a similar action to aqueous extract No. 3. Extract No. 5 did not present a clear antiviral effect. Instead, a stimulating effect was noticeable at a high concentration; i.e., it was a substance that stimulated cell growth. Differences in the dynamics of change increased along the series between infected and uninfected plates. It is interesting to note that substance No. 6 also presented a stimulating effect on cell growth, which was most pronounced at 3.3 mg/mL. Extract No. 7 presented an antiviral effect at concentrations of 0.37–3.33 mg/mL. The activity was concentration-dependent and a maximum effect was observed at 3.33 mg/mL. The *Artemisia cina* Berg. extract was toxic at a concentration of 10 mg/mL (Figure 1 and Figure S8). Previously, the three extracts of *Artemisia Annua* L. were evaluated against the cell line VeroE6 in vitro. The IC_50_ values obtained were as follows: VOP was 1587 µg/mL, BOP was713 µg/mL, and SOP was >2000 µg/mL (for the SOP extract, 50% growth inhibition was not achieved, even at the highest concentration tested) [18].

### 2.2. Evaluation of Toxicity In Vivo

*Artemisia cina* Berg. extracts No. 7, 3, and 4 at the maximum technically achievable dose, which was 2 g/kg of body weight of mice, did not lead to the death of laboratory animals. Therefore, it was not possible to determine the LD_50_ value. The results of the influence of extracts No. 7, 3, and 4 on the dynamics of the mice body weights with a single intragastric administration at a dose of 2 g/kg of body weight of mice are presented in Table 1.

The data presented in Table 5, Table 6 and Table 7 testify to the absence of the influence of tested complex substances on the body weight of mice following a single intragastric administration at a dose of 2 g/kg. Throughout the experiment, the general condition and behavior of the animals, as well as the manifestation of symptoms of intoxication, were monitored (Table 2 and Table 3).

On the first day after the introduction of the test and throughout the whole experiment using *Artemisia cina* Berg. extracts No. 7, 3, and 4, changes in the behavior, appearance, and motor activity of laboratory animals were observed. Fortunately, the high-dose administration of extracts No. 7, 3, and 4 did not cause the death of laboratory animals, and any significant changes in their behavior were observed.

The biochemical analysis of urine (in terms of the parameters of protein, glucose, and pH level), presented no deviations from the normal physiological values characteristic of laboratory mice (Table 8 and Table 9). In all experimental groups of animals, the content of erythrocytes in the urine slightly increased one day after the administration of the test *Artemisia cina* Berg. extracts. After a week, this indicator decreased somewhat; however, it remained elevated compared to the values for the control animals. In two experimental groups (No. 7, 3, and 4), there was a slight increase in ketones in the urine. Two weeks after the introduction of extracts No. 7, 3, and 4, a macroscopic examination of the internal organs of the laboratory animals was performed. The internal organs were removed, weighed, and the weight coefficients of the internal organs were determined (Table 10). A macroscopic picture of the internal organs of the laboratory animals, which were injected with extracts No. 7, 3, and 4, was not different from the picture of the internal organs of the control laboratory animals. The mass of the internal organs of mice that received extracts No. 7, 3, and 4 did not differ from the mass of the internal organs of the control mice (Table 10).

## 3. Materials and Methods

Sodium methoxide CH_3_ONa (98%) and sodium ethoxide C_2_H_5_ONa (96%) were obtained from Alfa Aesar cytisine standard (98%); methanol (99.9%), acetonitrile (99.8%), Santonin standard (99.5%) DMSO 99.5%, and TLC silica gel matrix with fluorescent indicator 254 nm, which were obtained from Sigma Aldrich (Darmstadt, Germany). Nitrosine blue tetrazolium (MTT) was bought from Sigma M2128. HCl 30 *v*/*v*%, H_2_SO_4_ 90 wt./*v*.%, NaOH 99.5%, NaClO_4_ 99.0%, acetic acid 99.0%, CH_3_COONa 99.5%, Hexane 99%, ethyl acetate 99.5%, ethanol 95% *v*/*v*, petroleum ether 99%, chloroform 99%, and filter-paper-grade blue tape were purchased from LTD LabChimprom (Almaty, Kazakhstan). Aqueous solutions were prepared using distilled water.

### 3.1. Collection and Preparation of Plant Raw Materials

The collection and preparation of plant raw materials was conducted in accordance with the WHO Guidelines on the Good Cultivation and Collection Practices (GACP) of medicinal plants, approved by the World Health Organization in 2003 [19]. The subject of our research was the aerial part of the medicinal plant raw materials of the herb wormwood, which were collected in August 2021 in the Turkestan region, Arys district, in the village of Dermen (GPS 42°31′12.4″ N 68°47′43.3″ E). The collection and preparation of the plant materials of Artemisia cina Berg. were performed in accordance with the Good Agricultural and Collection Practices For Medicinal Plants (GACP) in the summer period of 2021, during their mass-budding phase, in the village of Dermene, Turkestan region, the Republic of Kazakhstan. They were then identified by the Institute of Botany and Phytointroduction, Science Committee, Ministry of Education and Science of the Republic of Kazakhstan. A voucher sample (№01-04/257 from 15 August 2021) was deposited in the herbarium of the Institute of Botany and Phytointroduction, Almaty, Republic of Kazakhstan.

*Artemisia cina* Berg. is an endemic plant of the Republic of Kazakhstan. During its budding phase, the above-ground part of of Artemisia cina Berg. was cut with sickles at a height of 10–15 cm from the ground and placed into small shocks with butts facing outward, using the manual method of collection and cleaning within a time schedule ranging from 7.00 to 10.00. The grass was then dried at the enterprise “D.V. Sokolsky Institute of Fuel, Catalysis and Electrochemistry” JSC in the shade on special frames, at an ambient temperature of 28 ± 5 °C; the grass was then laid out in layers measuring 10–15 cm and periodically turned over. To develop the optimal technology and obtain various extracts, the extractant, method, and conditions for conducting the extraction process were then selected.

### 3.2. Obtaining Extracts from Plant Raw Materials

The extraction of *Artemisia cina* Berg. raw materials of tarragon wormwood was conducted using the method of fractional maceration. The extraction conditions were as follows: the extraction time was 30 min in an ultrasonic bath (ultrasonic power: 50 watts), and the temperature applied was in the range of 40 °C to 70 °C, depending on the extractant.

Extracts collected from plant raw materials were obtained using ultrasonic extraction methods. The extracts obtained using ultrasonic extraction were subjected to concentration (solvent distillation on a rotary evaporator) and lyophilization processes on a freeze dryer.

*Ultrasonic extraction* was performed using a PS-06A 0.6L digital ultrasonic cleaner. The *Artemisia cina* Berg. raw materials, having been precrushed to a particle size of 1 mm (with the size set by sieving through a sieve; the cell size being 1 mm), were placed in a glass container with a volume of 3 L. First, we filled a container with an alcohol–water solution and ensured the thorough mixing of the solution. Then, we placed this container in an ultrasonic bath set at a frequency of 40 kHz and a temperature of 30 °C for a duration of 1 h. To maintain constant stirring conditions during the extraction process, we attached a propeller stirrer to the top of the container. The ultrasound treatment should be performed for 30 min, followed by a 30 min pause. Repeat this cycle of work two times.

Subsequently, we filtered the extract that underwent ultrasound treatment and concentrated it using a rotary evaporator (Stuart RE400, Cole-Parmer Ltd., Altrincham, UK) under gentle conditions. We ensured that the temperature of the water bath did not exceed 65 °C, and continued the process until the volume was reduced to one-third of the original volume.

#### 3.2.1. Lyophilization of Thick Extracts

The method for producing dry extracts involved using a freeze dryer called Nova Dryer-HF100 (manufactured by Senova Biotech, Shanghai, China). Thick extracts were placed on specialized shelves located inside the freeze-dryer chamber for the lyophilization process. To monitor the temperature of the extracts, a PT100 sensor was inserted into one of the extracts. Several parameters were configured for the operation, including the prefreezing time, cold trap setting, prevacuum setting, and vacuum limit. The vacuum-control mode was selected, which allowed for further parameter adjustments. In this mode, such parameters as the upper and lower vacuum limits, initial drying time, pressure maintenance time, temperature, and final drying stage were set. Additionally, a temperature-keeping stage was adjusted to preserve the extracts following the lyophilization process until the device was turned off. Once all the drying stages were completed, an indicator light signaled the end of the process. The drain valve was then opened to release the pressure, and the fully dried sublimates were removed from the device.

#### 3.2.2. Selection of the Extractant

Dried and crushed raw materials were extracted with polar organic solvents (acetone, 50% acetone, ethyl alcohol, and aqueous solutions of the latter (50%, 70%, and 90%), water), butanol, ethyl acetate, propanol, 50% propanol, and chloroform, in addition to the nonpolar solvents hexane and benzene, while maintaining the consistency of the mass of raw materials (10 g), the volume of the solvent (50 mL), and the ratio of the raw materials and solvent (1:5), as well as the extraction time (2 days). The extract was quantitatively transferred into a porcelain cup with a diameter of 2–3 cm and evaporated in a water bath, after which they were brought to a constant mass in a drying cabinet.

#### 3.2.3. Selection of the Optimal Ratio of Extractants: Raw Material

Different volumes of solvent (50% ethyl alcohol) were added to several samples of plant raw materials of the same mass (5 g): 25, 30, 40, and 50 mL. At the same time, the constant factors of the extraction process were: extraction time (24 h) and temperature (20–28 °C).

The hot water extracts of *Artemisia cina* Berg. that were selected for antiviral tests were as follows:

(a) No. 2 extract was prepared as follows: 50 g of raw material of *Artemisia cina* Berg. Was extracted with 250 mL of petroleum ether; then, the petroleum ether was distilled off and extracted with 250 mL of hexane into the obtained meal;
(b) No. 3 extract, an aqueous extract of wormwood following fractional extraction, was prepared as follows: 50 g of *Artemisia cina* Berg. was sequentially extracted with petroleum ether, hexane, and ethyl alcohol. Subsequently, the remaining substances were extracted from the obtained meal with the help of 250 mL of hot water, and then the water was distilled from the obtained tincture (the extract was concentrated). Product yield: 4.91 g. The scheme of the extract is presented in Figure S1;
(c) No. 4 extract was prepared as follows: 10 g of *Artemisia cina* Berg. was infused in 200 mL of hot water for 2 h, and then water was distilled from the resulting tincture (the extract was concentrated). Product yield: 7.31 g. The scheme of the extract is presented in Figure S2;
(d) No. 5 extract was prepared as follows: 50 g of raw material of *Artemisia cina* Berg. was extracted with 250 mL of petroleum ether; then, the petroleum ether was distilled off and extracted with 250 mL of 96% alcohol into the obtained meal;
(e) No. 6 extract (using 40% ethanol) was prepared as follows: 50 g of *Artemisia cina* Berg. was infused in 250 mL of 40% alcohol solution, and then the water and alcohol were distilled from the resulting tincture (i.e., the extract was concentrated). Product yield: 5.12 g. The scheme of the extract is exhibited in Figure S3;
(f) No. 7 extract (*Artemisia cina* Berg., 1 tsp): A total of 5 g of wormwood was infused in 200 mL of hot water, and then the water was distilled from the resulting tincture (i.e., the extract was concentrated). Subsequently, 5 mL of hexane was added to the obtained concentrated extract to remove hexane-soluble toxic impurities. Product yield: 1.78 g. The scheme of the extract is illustrated in Figure S4.

#### 3.2.4. Gas Chromatography with a Mass Spectrometer

An Agilent 6890/5973 chromato-mass spectrometer was used. GL Science Inert Cap column: 25–30 m, 0.32 mm, 0.25 microns. The temperature of the evaporator was 260 °C. The carrier gas (helium) charged the column with a flow rate of 2.3 mL/min.

The temperature of the column was programmed as follows:

- Temperature: 80 °C for 1 min;
- Heating at a rate of 10 °C per minute to 250 °C, and held for 5 min at this temperature;
- Total analysis time: 23 min.

Operating conditions of the mass spectrometer:

- Quadrupole temperature: 150 °C;
- Source temperature: 230 °C;
- Interface temperature: 280 °C;
- The voltage of the ion multiplier: 1388 V;
- The polarity was positive.

The sample was entered using an autosampler in the amount of 1 µL. Prior to the analysis, the samples were dissolved in methanol.

#### 3.2.5. Separation of Extracts into Separate Fractions and Individual Components

The separation of the plant extracts into separate fractions was performed using column and thin-layer chromatography methods, according to the following methods. The following materials were used for thin-layer and column chromatography techniques: plates for thin-layer chromatography (Sigma Aldrich 60 matrix, with a size of 20 cm × 20 cm) with a fixed-silica-gel layer of Supelco 288624-60 Å silica gel powder, a glass chamber with a lid measuring 50 cm × 40 cm × 10 cm, and glass columns measuring 20 × 1.5 cm, 40 × 2 cm, and 60 × 3 cm; solvent systems chloroform: methanol in different volume ratios, hexane: ethyl acetate in different volume ratios, paper filter, microcapillary, and UV camera CAMAG UV Lamp 4 with a wavelength of 254 nm.

The TLC plate was removed from the chamber and left to dry completely. Then, the stain samples were examined in the UV cabinet chamber. When the separation was complete, each component appeared as vertically separated spots. Each spot had a retention coefficient (Rf) expressed as: Rf = distance traveled by the sample/distance traveled by the solvent.

#### 3.2.6. Method of Evaluation of Antiviral Activity

The culturing of the virus SARS-CoV-2 was conducted using a previously published method [20,21] at the premises of the Republican State Enterprise “National Center for Biotechnology”, the Ministry of Healthcare of the Republic of Kazakhstan, in accordance with the safety regulations of working with pathogenic viruses.

Six-well tablets were seeded with Vero E6 cells (for the titration of the SARS-CoV-2 virus). The sowing dose was 5 × 10^5^ cells per well. The tablets were incubated for 4 h. Ten-fold dilutions of the virus (SARS-CoV-2) were prepared. The dilutions were prepared in PBS+1% heat-activated horse serum (HI HS). For infection, the medium was removed from the wells; 0.2 mL of the corresponding inoculum was added to each well. The tablet was incubated at 37 °C for at least 1 h. Every 15 min, the tablet was shaken in order to distribute the inoculum evenly to the other half. Inoculates were removed from the wells. A total of 2 mL of agarized medium (full DMEM medium, with the addition of 1.5% fusible agarose) was introduced into the wells. The tablets were incubated for 3 days, periodically microscopizing the growing monolayers. Coronavirus CE manifests itself as plaques filled with dead rounded cells. Viral plaques were visualized by staining the tablets: 0.5 mL of MTT solution (3 mg/mL) was injected into each well in a 1X MEM medium (without additives). The tablets were incubated for 2 h at 37 °C in a CO_2_ incubator [18].

The production of viral preparations (stocks): VeroE6 cells were seeded in P100 Petri dishes in the amount of 2 × 10^6^ cells. Cultures were grown to 90% confluency (~8 × 10^6^ cells). To infect the monolayers and obtain viral preparations, a medium similar to complete medium was used; however, with the addition of 2% heat-inactivated serum (FBSHI serum, heated at 55 °C for 30 min). The standard virus production medium was changed to 2% FBSHI medium, after which the virus was added to the culture so that the multiplicity of infection (MOI) was 0.01. The culture dishes were incubated in a CO_2_ incubator for 72 h. The cultures were microscopically examined daily and monitored for the appearance of virus-induced cytopathic action (CPE) [18]. Medium obtained from infected cultures was collected 72 h after infection. The medium was then clarified using centrifugation, divided into 0.5 mL aliquots, and the virus was stored at −80 °C until use. For the determination of the virus titer, the limiting dilution method (Reed–Muench method) with modifications was used. VeroE6 cells were seeded in 96-well plates (37,500 cells per well). Serial dilutions of the SARS-CoV-2 virus were prepared. Phosphate buffered saline (PBS) supplemented with 1% heat-inactivated horse serum was used as a diluent. Eight 10-fold dilutions were produced, ranging from 1:10 to 1:108. One dilution occupied a long row on a 96-well plate (11 wells). In all the experiments, row 12 of the plate was left uninfected; this row was used as a growth control for the uninfected monolayer. The medium was then removed from the wells. Row 12 was filled with 150 μL of medium with 2% FBSHI. Wells at the intersection of rows H–A and 1–11 were filled with dilutions of the virus, at 100 μL per well. The plate was incubated for 1 h with occasional shaking to mix the inoculums. Then, viral inoculums (from rows H–A × 1–11) were removed and 150 μL of medium with 2% FBSHI was added to the wells. The plates were incubated in a CO_2_ incubator at 37 °C for 3–4 days until a visible virus-induced cytopathic effect appeared [18].

In all plates, the number of wells with a visible virus-induced cytopathic effect was counted in each row. The results were processed according to the Reed–Muench scheme [22]. The coronavirus titer was expressed in infectious units TCID50 (a unit of 50% probability of tissue culture infection). The determination of 50% inhibitory concentration (IC_50_) was achieved. The cytotoxic effect of the extracts was measured by determining the half-maximal inhibitory concentration IC_50_ (i.e., the concentration that inhibited the growth rate of the culture compared to the control by 2 times, and reduced the density of the cell monolayer by 50%). VeroE6 cells were seeded in wells of a 96-well plate at 20,000 cells per well. The plates were then incubated overnight. The subsequent day, weighed portions of the studied extracts (200 mg) were dissolved in dimethyl sulfoxide (DMSO) to obtain solutions with a concentration of 200 mg/mL. The resulting DMSO solution was then diluted 100 times with the culture medium to obtain samples at a concentration of 2000 μg/mL (this was the sample used to fill row H). Dilutions (of test extracts) were applied to long rows of a 96-well plate (samples were applied to the wells of rows 1–10. Rows 11–12 did not contain extracts; these rows were used to control the growth of a healthy monolayer without exposure to the test substances). The procedure was as follows: the growth medium was removed from the wells (rows H–A × 1–10) and 100 µL of the medium was added to rows A–G. A total of 150 μL per sample well was added to row H to fill it (i.e., a solution of the extract in the medium at a concentration of 2000 μg/mL). Then, using an 8-channel pipette, 50 µL of the sample was transferred to a parallel row (from H to G, from G to F, etc.); with each transfer process, the contents of the wells were mixed. The plates were then incubated in a CO_2_ incubator for 3 days. The state of the cells in culture was monitored using the microscopy method.

### 3.3. MTT Assay

To quantify the density of living cells in the monolayers, a colorimetric test with nitrosine blue tetrazolium staining was used. A total of 20 μL of MTT solution (3 mg/mL) in a medium without serum was added to the wells of culture plates. The plate was then incubated for a period of 3 h. The medium was quantitatively removed from the wells without disturbing the integrity of the stained monolayers. Formazan was dissolved in 100 μL of DMSO (with the addition of 1% acetic acid). The optical density in the wells was determined on a tablet photometer at a wavelength of 595 nm [23]. For each extract, the experiment was performed in duplicate. An estimate of the IC50 value was calculated from the absorbance data using a four-parameter nonlinear regression. The results were then processed using the GraphPadPrism program (GraphPadInc., San Diego, CA, USA) [23].

### 3.4. Evaluation of Toxicity of Plant Extracts In Vivo

To estimate the acute toxicity of substances No. 7, 3, and 4, the contents of each vial were administered to laboratory CD-1 mice of both sexes once, intragastrically, at the maximum technically achievable dose (which was 2 g/kg of animal body weight). The dose of the test substance was calculated based on the body weight of the laboratory animal. Taking into account the body weight of the mice, the amount of substances was calculated for injection and prepared immediately prior to injection into the test system. In order to perform the intragastric administration of substances to the laboratory mice, the contents of each vial were diluted in drinking water, after which the resulting solutions were administered ex tempore. The control animals were then injected intragastrically with drinking water

The method of performing the toxicological study of *Artemisia cina* Berg. extracts No. 7, 3, and 4 in vivo with a single application (acute toxicity study) was applied in accordance with a previous study [24].

The study of acute toxicity included a one-time administration (intragastrically) to outbred mice of the CD-1 line into their stomach, and in the maximum technically achievable dose of 2 g/kg of animal body weight. White laboratory mice of the CD-1 line of both sexes weighing 30 ± 3 g (females) and 33 ± 3 g (males) were used. The laboratory animals were obtained from the vivarium of the Republican State Enterprise “National Center for Biotechnology”. Groups were formed by taking into account the receipt of statistically significant results of 6 animals in each study group. In the experiments, 48 mice of the CD-1 line were used, with 12 mice (6 females and 6 males) per group across 3 experimental groups and 1 control group. The total duration of the observation of animals in the study of acute toxicity was 2 weeks; on the first day, following the introduction of substances, the animals were under continuous observation [7]. Measured parameters: general condition of animals, behavioral features, motor activity, the presence and nature of seizures, reactions to stimuli, the frequency and depth of respiratory movements, the condition of hair and skin, the color of mucous membranes, the quantity and consistency of fecal masses, and change in body weight.

After a total of both 24 h and 1 week following the introduction of substances No. 7, 3, and 4, a biochemical analysis of their urine was performed, using indicator test strips for the qualitative and semiquantitative determinations of erythrocytes, ketones, protein, glucose, and pH level (Uripolian-5A, Biosensor AN, Russia). It was registered under the terms of development of the possible intoxication and death of animals. At the end of the experiment (two weeks following the introduction of substances No. 7, 3, and 4), a macroscopic study of the internal organs of animals was performed with the determination of the masses of the internal organs. The mean lethal concentration (LD_50_) in the event of the death of animals was calculated using the Kerber method [7].

The control group and the treated animals were of the same sex and age, and were received simultaneously from the same nursery and kept in similar conditions. The conditions of keeping and feeding animals corresponded to the established rules [7]. Due to the fact that the action of the pharmacological preparation depends on the physiological state of the animals (which changes under the influence of a number of external factors), all studies were performed in the same time of day (morning to afternoon). Regarding the mode of administration, method of administration, and dose level, No. 7, 3, and 4 were administered once, intragastrically. Intragastric administration was conducted on animals that were awake (nonanesthetized). The animal was fixed by the root of the tail, placed on the table, and covered with a towel. Holding the tail with the hand, the animal was lightly pressed against the table with the palm of the hand. The animal was taken by the skin in the region of the occiput with the thumb and forefinger; the cheeks were stretched, and the mouth was opened. The animal was placed head up. The probe was introduced along the posterior wall of the pharynx and advanced along the esophagus until it entered the stomach. The substance was administered with a syringe after the probe entered the stomach. The control group of animals was injected with the appropriate solvent (drinking water) in the same volume (0.5 mL) and according to the same scheme (i.e., once) as the studied substances No. 7, 3, and 4. The number of experimental animals was 48 CD-1 mice,: 12 mice, split into 6 females and 6 males in each group across 3 experiments and 1 control. Groups of animals for the experiment to determine the acute toxicity of substances No. 7, 3, and 4 were distributed as follows:

(1) Group 1 (6 female mice): laboratory female mice in this study group were injected once, intragastrically, with the substance No. 7 *Artemisia cina* Berg. in the maximum technically achievable dose of 2 g/kg of body weight of mice;
(2) Group 2 (6 female mice): laboratory female mice in this study group were injected once, intragastrically, with the substance No. 3 *Artemisia cina* Berg. in the maximum technically achievable dose of 2 g/kg of body weight of mice;
(3) Group 3 (6 female mice): laboratory female mice in this study group were injected once, intragastrically, with the substance No. 4 *Artemisia cina* Berg. in the maximum technically achievable dose of 2 g/kg of body weight of mice;
(4) Group 4 (6 female mice): laboratory female mice in this study group were injected intragastrically with the appropriate solvent (drinking water) in the same volume (0.5 mL) and according to the same scheme (once) as the test substance (manipulation control).
(5) Group 5 (6 male mice): laboratory male mice in this study group were injected once, intragastrically, with the substance No. 7 *Artemisia cina* Berg. in the maximum technically achievable dose of 2 g/kg of body weight of mice;
(6) Group 6 (6 male mice): laboratory male mice in this study group were injected once, intragastrically, with the substance No. 3 *Artemisia cina* Berg. in the maximum technically achievable dose of 2 g/kg of body weight of mice;
(7) Group 7 (6 male mice): laboratory male mice in this study group were injected once, intragastrically, with the substance No. 4 *Artemisia cina* Berg. in the maximum technically achievable dose of 2 g/kg of body weight of mice;
(8) Group 8 (6 female mice): laboratory female mice in this study group were injected intragastrically with the appropriate solvent (drinking water) in the same volume (0.5 mL) and according to the same scheme (once) as the test substance (handling control).

### 3.5. Ethical Aspects

The research of laboratory animals was conducted in accordance with the generally accepted ethical standards for the treatment of animals, based on the standard operating procedures that comply with the rules adopted by the European Convention for the Protection of Vertebrate Animals Used for Research and Other Scientific Purposes. The protocol for conducting research on the project “Development of highly effective medicinal substances from plant raw materials with antiviral activity against COVID-19 and similar viral infections” was approved by the Local Ethical Commission of the National Center for Biotechnology (Astana, Kazakhstan), 3 May 2022, Protocol No. 6.

### 3.6. Statistical Analysis of Data

The statistical processing of the results was performed using the Microsoft Excel 97 program. Intergroup differences were assessed using the nonparametric Mann–Whitney U test. In the case of pairwise related groups, the nonparametric Wilcoxon test was applied.

## 4. Conclusions

A comparative analysis was conducted on six extracts of *Artemisia cina* Berg., utilizing various extraction conditions. Among the methods tested, ultrasonic maceration was identified as the optimal technology for extracting *Artemisia cina* Berg. extract from the raw wormwood material. Hot water and ethanol were employed as extractants, with a plant-to-extractant ratio of 1:5 after fractional extraction. The primary component in the resulting extracts was observed to be a-santonin. Notably, extracts No. 3 and 7 exhibited a santonin content of 66%, while extract No. 4 (obtained using a 1:10 ratio) contained santonin at a 39% lower concentration. All identified compounds in the aqueous extract of *Artemisia cina* Berg. were confirmed to be santonin through mass spectrometry, displaying a characteristic signal with *m*/*z* 246. Regarding the antiviral effect, extract No. 3 demonstrated a broader range of concentrations where cellular protection was evident compared to extract No. 4.. To determine the maximum technically achievable dose of extracts No. 3 and 4, IC_50_ values at concentrations of 200 and 75 ug/mL were exhibited. Additionally, extract No. 3 exhibited lower toxicity levels compared to extract No. 4. To determine the maximum technically achievable dose of *Artemisia cina* Berg., the mice were administered a dosage of 2 g/kg of their body weight, resulting in a 100% survival rate, confirming that extracts No. 7, 3, and 4 of *Artemisia cina* Berg. can be classified as substances with low toxicity levels.

## Figures and Tables

**Table 1:** Dilution of the plant extract.

Row of the 96-Well Plate	Extract Concentration, mg/mL
A	0.004
B	0.01
C	0.04
D	0.12
E	0.37
F	1.11
G	3.33
H	10.0

**Table 2:** Component composition of *Artemisia cina* Berg. extract No. 3.

No.	Main Compounds	Relative Amount, %	R_t_, Minutes
1	α-santonin	66.3	18.23
2	6-nitro-2-phenyl-4-quinolinol	8.0	18.33
3	3,5,5-trimethylcyclohexylisophosphofloride	6.7	9.98
4	Lumisantonin	3.7	16.36
5	2,3-Dihydro-4H-pyran-4-one	3.2	5.27

**Table 3:** Component composition of extract *Artemisia cina* Berg. No. 4.

No.	Main Compounds	Relative Amount, %	R_t_, Minutes
1	α-santonin	39.39	18.22
2	Butanoic acid	10.05	12.24
3	Anhydro-β-retinol	3.28	21.02

**Table 4:** Component composition of extract *Artemisia cina* Berg. No. 7.

No.	Main Compounds	Relative Amount, %	R_t_, Minutes
1	α-santonin	66.35	18.22
2	2,3-Dihydro-4H-pyran-4-one	4.90	5.42
3	4-Nonanone, 7-ethyl-	5.90	9.98
4	Lumisantonin	2.58	16.36

**Table 5:** The effect of *Artemisia cina* Berg. extracts No. 7, 3, and 4 on the body weight of mice with a single intragastric administration at a dose of 2 g/kg of body weight of mice.

Study Groups, Sex, Number	Initial Body Weight of Mice, g	Body Weight of Mice 1 Week after the Administration of the Test Substances, g	Body Weight of Mice 2 Weeks after the Administration of the Test Substances, g
1st group, No. 7 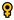 , n = 6	29.3 ± 2.1	31.0 ± 2.2	32.7 ± 2.0
	*p* = 0.6970	*p* = 0.7548	*p* = 0.6317
2nd group, No. 3, 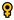 , n = 6	29.5 ± 1.1	31.9 ± 0.9	33.4 ± 0.9
	*p* = 0.8836	*p* = 0.9518	*p* = 0.7700
3rd group, No. 4, 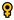 , n = 6	29.4 ± 1.8	32.4 ± 2.0	34.6 ± 1.8
	*p* = 0.8763	*p* = 0.9196	*p* = 0.8907
4th group, control, 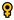 , n = 6	29.9 ± 2.5	32.1 ± 2.5	34.2 ± 2.3
29.9 ± 2.5	33.4 ± 1.4	35.2 ± 1.2	36.3 ± 0.9
	*p* = 0.4729	*p* = 0.7145	*p* = 0.3984
6th group, No. 3, 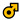 , n = 6	33.7 ± 2.1	35.8 ± 1.9	37.3 ± 1.4
	*p* = 0.5115	*p* = 0.5978	*p* = 0.4195
7th group, No. 4, 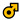 , n = 6	32.4 ± 2.7	36.4 ± 1.2	37.3 ± 1.2
	*p* = 0.8926	*p* = 0.2559	*p* = 0.4010
8th group, control, 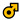 , n = 6	32.0 ± 1.3	34.6 ± 1.0	35.2 ± 2.0

Notes: 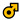 is a symbol indicating males; 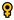 is a symbol indicating females; *p* is the significance level, and a *p* < 0.05 indicated statistically significant differences compared to the corresponding values in the control group of animals.

**Table 6:** Influence of *Artemisia cina* Berg. extracts No. 7, 3, and 4 on the condition of female mice with a single intragastric injection at a dose of 2 g/kg of body weight of mice for two weeks.

Investigated Parameter	Group of Animal, n = 6, 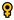			
	1st Group, No. 7	2nd Group, No. 3	3rd Group, No. 4	4th Group, Control
Intensity and nature of motor activity	The mice are active. The coordination of movements is not disturbed			
The presence and nature of seizures	Absent			
Condition of hair and skin	No changes detected (wool is white, clean, smooth)			
Condition and color of mucous membranes	No changes found or observed			
Reaction to sound, pain stimuli	Adequately react			
Animal death	0			
Urination (color of urine)	No changes found			
Defecation	No changes found			

Notes: 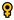 is a symbol indicating females.

**Table 7:** Influence of sum of substances in *Artemisia cina* Berg. extracts No. 7, 3, and 4 on the condition of male mice with a single intragastric injection at a dose of 2 g/kg of body weight of mice for two weeks.

Investigated Parameter	Group of Animal, n = 6, 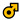			
	5th Group, No. 7	6th Group, No. 3	7th Group, No. 4	8th Group, Control
Intensity and nature of motor activity	The mice are active. The coordination of movements is not disturbed			
The presence and nature of seizures	Absent			
Condition of hair and skin	No changes detected (wool is white, clean, smooth)			
Condition and color of mucous membranes	No changes found or observed			
Reaction to sound, pain stimuli	Adequately react			
Animal death	0			
Urination (color of urine)	No changes found			
Defecation	No changes found			

Notes: **
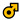
** is a symbol indicating males.

**Table 8:** Influence of *Artemisia cina* Berg. extracts No. 7, 3, and 4 on the biochemical parameters of urine one day after the introduction of experimental extracts with a single intragastric injection at the dose of 2 g/kg of body weight of mice.

Investigated Group	Researched Parameters				
	Erythrocytes, Units/µL	Ketones, mmol/L	Protein, g/L	Glucose, mmol/L	pH
1st group, No. 3, 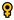 , n = 6	1/6—10 unit/μL 5/6—50 unit/μL	6/6—0.5 mmol/L	4/6—0.1 g/L 2/6—0.3 g/L	6/6—negative	6.0 ± 0.0 (6/6–6.0)
2nd group, No. 3, 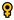 , n = 6	6/6—50 unit/μL	6/6—0.5 mmol/L	4/6—0.1 g/L 2/6—0.3 g/L	6/6—negative	6.0 ± 0.0 (6/6–6.0)
3rd group, No. 4, 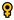 , n = 6	4/6—25 unit/μL 2/6—50 unit/μL	6/6—negative	2/6—0.1 g/L 4/6—0.3 g/L	6/6—negative	6.0 ± 0.0 (6/6–6.0)
4th group, control, 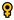 , n = 6	2/6—negative 4/6—10 unit/μL	6/6—negative	3/6—0.1 g/L 3/6—0.3 g/L	6/6—negative	6.0 ± 0.0 (6/6–6.0)
5th group, No. 7, 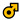 , n = 6	1/6—10 unit/μL 1/6—25 unit/μL 4/6—50 unit/μL	6/6—0.5 mmol/L	4/6—0.1 g/L 2/6—0.3 g/L	6/6—negative	6.0 ± 0.0 (6/6–6.0)
6th group, No. 3, 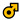 , n = 6	3/6—25 unit/μL 3/6—50 unit/μL	2/6—negative 4/6—0.5 mmol/L	3/6—0.1 g/L 3/6—0.3 g/L	6/6—negative	6.0 ± 0.0 (6/6–6.0)
7th group, No. 4, 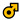 , n = 6	4/6—25 unit/μL 2/6—50 unit/μL	6/6—negative	2/6—0.1 g/L 4/6—0.3 g/L	6/6—negative	6.0 ± 0.0 (6/6–6.0)
8th group, control, 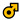 , n = 6	3/6—negative 3/6—10 unit/μL	6/6—negative	3/6—0.1 g/L 3/6—0.3 g/L	6/6—negative	6.0 ± 0.0 (6/6–6.0)

Notes: 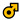 is a symbol indicating males; 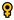 is a symbol indicating females; *p* is significance level, and a *p* < 0.05 indicates statistically significant differences compared to the corresponding values in the control group of animals; n is the number of animals in the group.

**Table 9:** Influence of *Artemisia cina* Berg. extracts No. 7, 3, and 4 on the biochemical parameters of urine 1 week after the consumption of No. 7, 3, and 4 with a single intragastric injection at the dose of 2 g/kg of body weight of mice.

Investigated Group	Essential Physiological Parameters				
	Erythrocytes, Units/µL	Ketones, mmol/L	Protein, g/L	Glucose, mmol/L	pH
1st group, No. 7, 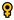 , n = 6	2/6—10 unit/μL 2/6—25 unit/μL 2/6—50 unit/μL	6/6—0.5 mmol/L	4/6—0.1 g/L 2/6—0.3 g/L	6/6—negative	6.0 ± 0.0 (6/6–6.0)
2nd group, No. 3, 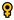 , n = 6	4/6—10 unit/μL 1/6—25 unit/μL 1/6—50 unit/μL	6/6—0.5 mmol/L	4/6—0.1 g/L 2/6—0.3 g/L	6/6—negative	6.0 ± 0.0 (6/6–6.0)
3rd group, No. 4, 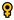 , n = 6	3/6—10 unit/μL 3/6—25 unit/μL	6/6—negative	1/6—0.1 g/L 5/6—0.3 g/L	6/6—negative	6.0 ± 0.0 (6/6–6.0)
4th group, 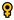 , n = 6	4/6—negative 2/6—10 unit/μL	6/6—negative	2/6—0.1 g/L 4/6—0.3 g/L	6/6—negative	6.0 ± 0.0 (6/6–6.0)
5th group, No. 7, 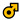 , n = 6	5/6—10 unit/μL 1/6—25 unit/μL	6/6—0.5 mmol/L	4/6—0.1 g/L 2/6—0.3 g/L	6/6—negative	6.0 ± 0.0 (6/6–6.0)
6th group, No. 3, 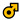 , n = 6	5/6—10 unit/μL 1/6—50 unit/μL	2/6—negative 4/6—0.5 mmol/L	4/6—0.1 g/L 2/6—0.3 g/L	6/6—negative	6.0 ± 0.0 (6/6–6.0)
7th group, No. 4, 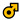 , n = 6	3/6—10 unit/μL 3/6—25 unit/μL	6/6—negative	2/6—0.1 g/L 4/6—0.3 g/L	6/6—negative	6.0 ± 0.0 (6/6–6.0)
8th group, control, 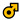 , n = 6	4/6—negative 2/6—10 unit/μL	6/6—negative	2/6—0.1 g/L 4/6—0.3 g/L	6/6—negative	6.0 ± 0.0 (6/6–6.0)

Notes: 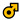 is a symbol indicating males; 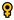 is a symbol indicating females; *p* is significance level, and a *p* < 0.05 indicates statistically significant differences compared to the corresponding values in the control group of animals; n is the number of animals in the group.

**Table 10:** Influence of *Artemisia cina* Berg. extracts No. 7, 3, and 4 on the weight of the internal organs of mice with a single intragastric injection at the maximum technically achievable dose of 2 g/kg of body weight of mice.

Investigated Group	Weight of Internal Animals Organs, g						
	Brain	Heart	Lungs	Liver	Spleen	Kidneys	Gonads
1st group, No. 7, 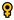 , n = 6	0.44 ± 0.0207 *p* = 0.3090	0.163 ± 0.0188 *p* = 0.7740	0.245 ± 0.0190 *p* = 0.1595	1.68 ± 0.0791 *p* = 0.2223	0.198 ± 0.0130 *p* = 0.8804	0.19 ± 0.0146 *p* = 0.2551	0.0176 ± 0.0008 *p* = 0.9560
2nd group, No. 3, 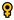 , n = 6	0.45 ± 0.0088 *p* = 0.2180	0.15 ± 0.0104 *p* = 0.2781	0.265 ± 0.0361 *p* = 0.7717	1.815 ± 0.0944 *p* = 0.6229	0.186 ± 0.0105 *p* = 0.5616	0.198 ± 0.0071 *p* = 0.2907	0.0187 ± 0.0012 *p* = 0.5011
3rd group, No. 4, 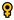 , n = 6	0.45 ± 0.0176 *p* = 0.5598	0.17 ± 0.0148 *p* = 0.8722	0.27 ± 0.0203 *p* = 0.8412	2.11 ± 0.0818 *p* = 0.2958	0.20 ± 0.0122 *p* = 0.9774	0.21 ± 0.0063 *p* = 0.6120	0.018 ± 0.0007 *p* = 0.8967
4th group, control, 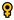 , n = 6	0.47 ± 0.0121	0.17 ± 0.0098	0.276 ± 0.0071	1.91 ± 0.1607	0.202 ± 0.0259	0.21 ± 0.0099	0.018 ± 0.0011
5th group, No. 7, 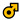 , n = 6	0.39 ± 0.0167 *p* = 0.9226	0.18 ± 0.0131 *p* = 0.7860	0.24 ± 0.0132 *p* = 0.4870	1.88 ± 0.0861 *p* = 0.6666	0.2069 ± 0.0132 *p* = 0.2501	0.22 ± 0.0050 *p* = 0.1597	0.102 ± 0.0026 *p* = 0.4869
6th group, No. 3, 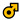 , n = 6	0.39 ± 0.0162 *p* = 0.8859	0.17 ± 0.0105 *p* = 0.5547	0.27 ± 0.0330 *p* = 0.7836	1.88 ± 0.1745 *p* = 0.7810	0.21 ± 0.0150 *p* = 0.3370	0.22 ± 0.0048 *p* = 0.2061	0.102 ± 0.0018 *p* = 0.4140
7th group, No. 4, 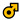 , n = 6	0.397 ± 0.0073 *p* = 0.7855	0.17 ± 0.0076 *p* = 0.5711	0.25 ± 0.0109 *p* = 0.6138	1.99 ± 0.0865 *p* = 0.7212	0.208 ± 0.0129 *p* = 0.2638	0.22 ± 0.0025 *p* = 0.3128	0.11 ± 0.0023 *p* = 0.2694
8th group, control, 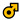 , n = 6	0.39 ± 0.0174	0.18 ± 0.0053	0.26 ± 0.0189	1.94 ± 0.1176	0.23 ± 0.0113	0.23 ± 0.0107	0.105 ± 0.0031

Notes: 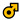 is a symbol indicating males; 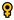 is a symbol indicating females; *p* is significance level, and a *p* < 0.05 indicates statistically significant differences compared to the corresponding values in the control group of animals; n is the number of animals in the group.

## Data Availability

Not applicable.

## Supplementary Materials

The following supporting information can be downloaded at: https://www.mdpi.com/article/10.3390/molecules28145413/s1, Figure S1: Block diagram of *Artemisia cina* Berg. extract production No.3; Figure S2: Block diagram for No. 4 extract production; Figure S3: Block diagram of *Artemisia cina* Berg. extract No. 6 extract; Figure S4: Block diagram of *Artemisia cina* Berg. extract No. 7; Figure S5: GC-chromatogramm of diluted Artemisia extract N3; Figure S6: GC-chromatogramm of diluted Artemisia extract N4; Figure S7: GC-chromatogramm of diluted Artemisia extract N7; Figure S8: Photographs of 96-well plates: not infected (positive control) and SARS-CoV-2 infected sample for dilutions of Artemisia extract: (A) N2; (B) N3; (C) N4; (D) N5; (E) N6; (F) N7; Table S1: Qualitative analysis of plant raw materials of wormwood (*Artemisia cina* Berg.) and annual wormwood.
