# Supplementary material for: An Estimation of the Antiviral Activity and Toxicity of Biologically Active Substances Obtained from the Raw Materials of Artemisia cina Berg. In Vitro and In Vivo
Source: Molecules. 2023 Jul 14;28(14):5413. doi: 10.3390/molecules28145413 (PMC10384809; doi:10.3390/molecules28145413)
Supplement: Supplementary file 1 [file molecules-28-05413-s001.zip › molecules-2406177-supplementary.pdf]

ESTIMATION OF ANTIVIRAL ACTIVITY AND TOXICITY OF BIOLOGICALLY ACTIVE SUBSTANCES FROM THE RAW MATERIALS OF *ARTEMISIA CINA* BERG. IN VITRO AND IN VIVO

Zhurinov M.Zh[1]., Berillo D.A.[*2.3] Bazhykova K.B[4]., Rakhimov K.D[3], and Bekezhanova T.S[*3]

1. «D.V. Sokolsky Institute of Fuel, Catalysis and Electrochemistry » JSC
2. Department of chemistry and biochemical engineering, Institute of Chemical and Biological Technologies (IHBT), Satbayev University Almaty 050013, Kazakhstan
3. NCJSC Asfendiyarov Kazakh National Medical University
4. Al-Farabi Kazakh National University

Correspondence: e-mail d.berillo@satbayev.university and bekezhanovat@kaznmu.kz;

Methods

Determination of technological and pharmacopoeial quality parameters of medicinal crude plant

Determination of such quality parameters of medicinal plant materials (MPM) as specific gravity, extractant absorption coefficient, extractive substances, weight loss during drying, total ash and ash insoluble in 10% hydrochloric acid makes it possible to predict the optimal method and conditions for extracting biologically active substances and normalize the technological process of obtaining a pharmacopoeial quality extract.

Determination of specific gravity.

5.0 g of raw materials (exact weight) were placed in a measuring flask with a capacity of 100 cm3, filled with 2/3 volume purified water and kept in a boiling water bath for 1.5 – 2 hours, periodically stirred to remove air. After that, the flask was cooled to 20 °C, the volume was brought to the mark with purified water. The flask was weighed and its mass was determined with raw materials and water. The weight of the flask with water was preliminarily determined. The specific gravity was calculated by the formula (1):

$$d_y = \frac{Pd}{P+G-F} \qquad (1)$$

where P - the mass of absolutely dry raw materials, g;

d - the density of water, g/cm3 (d = 0.9982 g/cm3).

G - the mass of the flask with water, g;

F - the mass of the flask with water and raw materials, g;

Determination of the extractant absorption coefficient (EAC).

5.0 g of crushed raw materials (exact weight) were placed in measuring cylinders and filled with an extractant (alcohol 30%, 50%, 70%, 96% and purified water) so that the raw material was completely covered, and left for several hours. Then the raw materials were filtered through a paper filter into another measuring cylinder and the amount of the extracted agent was recorded.

The extractant absorption coefficient was calculated according to the formula (2):

$$X = \frac{V - V_1}{P} \quad (2)$$

where V is the volume of the extractant with which the raw material was filled, cm 3;

V 1 – volume of extractant obtained after absorption of raw materials, ml;

P is the mass of dry raw materials.

Determination of extractive substances

The protocol was carried out according to State Pharmacopoeia of the Republic of Kazakhstan. About 3.0 g (exact weight) of raw materials crushed to 1 mm were placed in a conical flask with a capacity of 200-250 cm3, 50 cm3 of solvent (alcohol) was added 30 %, 50 %, 70 %, 96 % and purified water), the flask was closed with a stopper, weighed (with an accuracy of 0.01 g) and left for one hour. Then the flask was connected to a reverse refrigerator, heated in a water bath, maintaining a low boil for 2 hours. After cooling, the flask was weighed again, closed in advance with the same stopper, and the loss in mass was filled with solvent. The contents of the flask were shaken and filtered through a dry paper filter into a dry flask with a capacity of 150-200 cm3. 25 cm3 filtrates were pipetted into a pre-dried at a temperature of 100-105 °C to a constant weight and accurately weighted porcelain cup with a diameter of 7-9

cm and evaporated in a water bath to dry. The cup with the residue was dried at a temperature of 100-105 ° C to a constant weight, then cooled for 30 minutes in a desiccator with anhydrous calcium chloride and immediately weighed. The content of extractive substances (X %) in terms of absolutely dry raw materials according to the formula (3):

$$X = \frac{m*200*100}{m_1*(100-W)} \quad (3)$$

where m is the mass of the dry residue, g;

m1 – mass of raw materials, g;

W - is the mass loss during drying, %.

Determination of mass loss during drying (State Pharmacopoeia of the Republic of Kazakhstan, vol.1, 2.2.32.)

The analytical sample of raw materials was crushed to a particle size of about 10 mm, mixed and five samples weighing 1,000 g were taken. Each suspension was placed in a pre-dried and weighed together with the lid of the bux and placed in a drying cabinet heated to 100-105 ° C. The first weighing of herbs was carried out after 2 hours. Drying was carried out to a constant mass.

Determination of total ash (State Pharmacopoeia of the Republic of Kazakhstan, vol.1, 2.4.16.)

A quartz or platinum crucible was heated at a red knee for 30 minutes, cooled in a desiccator and weighed. 1.00 g of powdered medicinal plant raw material was placed in a crucible and evenly distributed over the bottom of the crucible. Dried at a temperature from 100 ° C to 105 ° C for 1 hour and then burned to a constant mass in a muffle furnace at a temperature of 600 ± 25 ° C, cooling the crucible in the desiccator after each combustion.

Determination of ash insoluble in hydrochloric acid (State Pharmacopoeia of the Republic of Kazakhstan, vol.1, 2.4.16.)

The ash insoluble in hydrochloric acid is the residue obtained after the extraction of the total ash, 15 ml of water and 10 ml of hydrochloric acid were added, the crucible was covered with a watch glass, the mixture was gently

boiled for 10 minutes, and then cooled. Filtered through an ashless filter, washed with hot water to a neutral pH value of the filtrate, dried, and then calcined to red, cooled in a desiccator and weighed. Calcination was carried out to a constant mass.

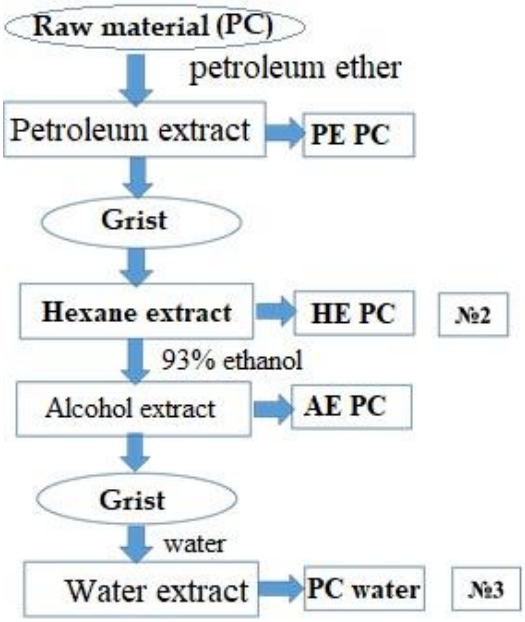

Figure S1 – Block diagram of *Artemisia cina Berg.* extract production No.3

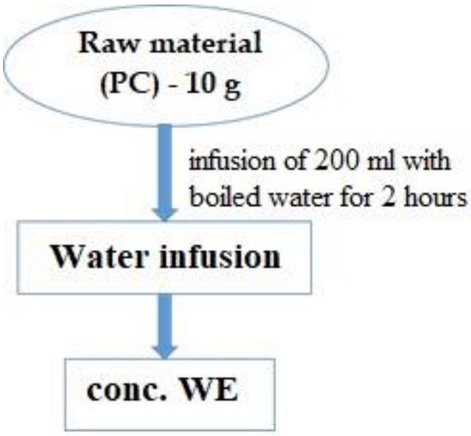

Figure S2 – Block diagram for No. 4 extract production

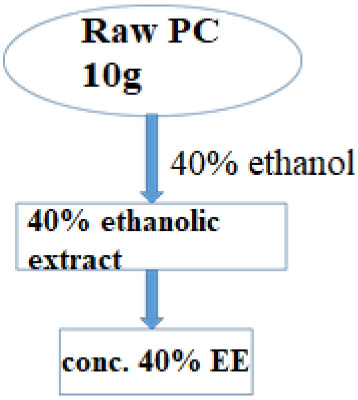

Figure S3 – Block diagram of *Artemisia cina Berg.* extract No. 6 extract production

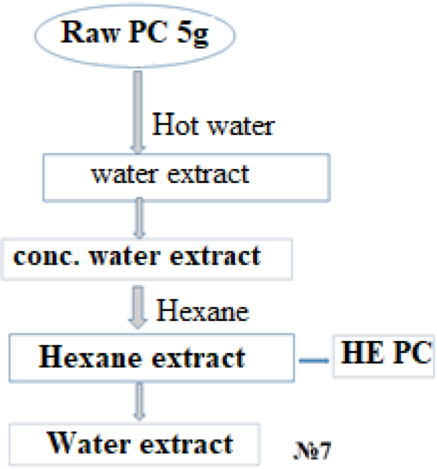

Figure S4– Block diagram of *Artemisia cina Berg.* extract №7

```
File        :C:\msdchem\1\DATA\Kataliz\VODAPC15.D
Operator    : Lyapunov
Acquired    :  9 Jun 2022  11:02     using AcqMethod Kataliz.M
Instrument  :   Instrument #1
Sample Name: voda pc 1/5     №3
Misc Info   : 1 mcl diluted 1/100 methanol
Vial Number: 1
```

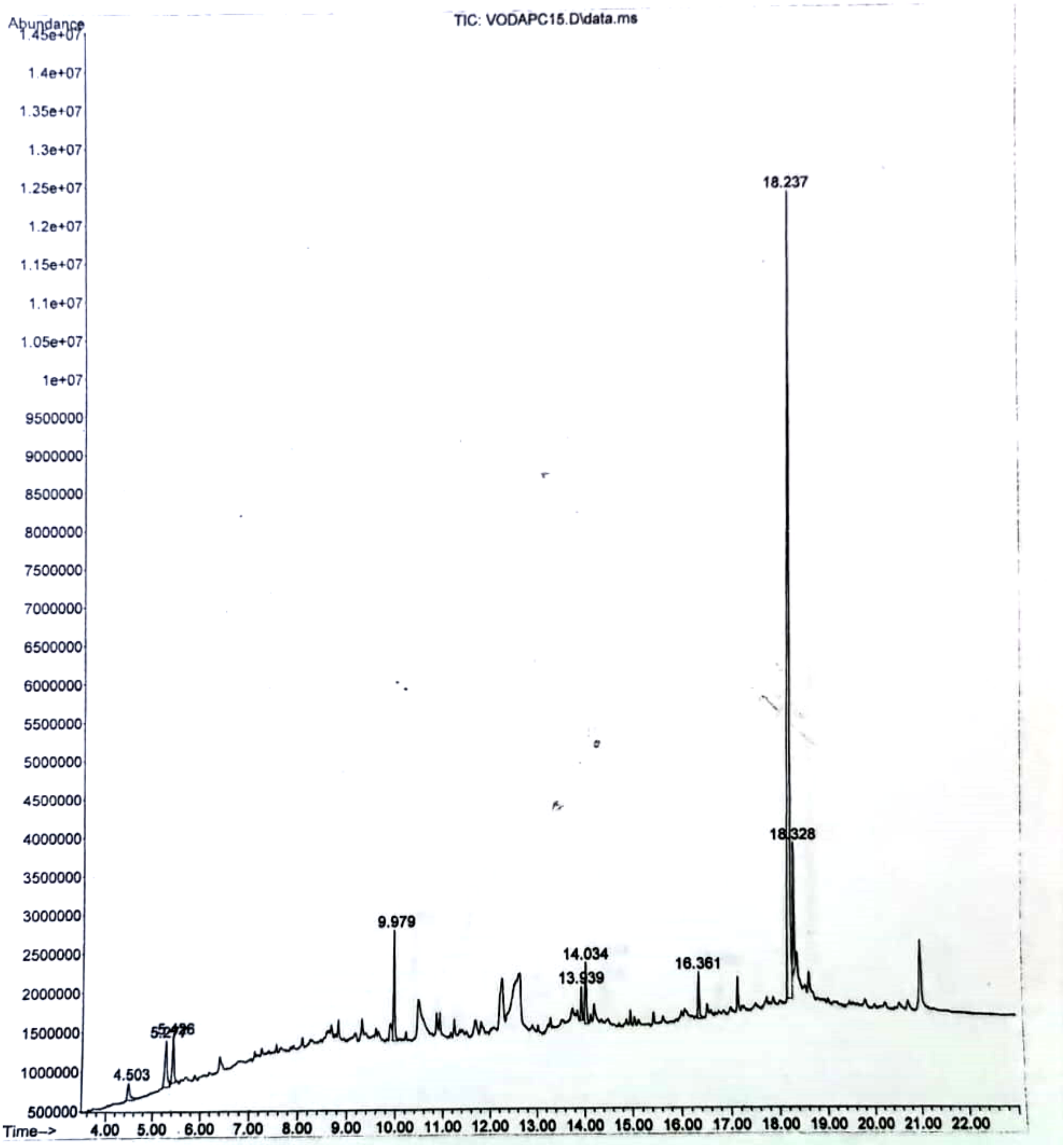

Figure S5 GC-chromatogramm of diluted Artemisia extract N3

```
File          :C:\msdchem\1\DATA\Kataliz\BSB\VODAPC110.D
Operator      : Lyapunov
Acquired      :  9 Jun 2022  11:31     using AcqMethod Kataliz.M
Instrument :    Instrument #1
Sample Name: voda pc 1/10        No4
Misc Info  : 1 mcl diluted 1/100 methanol
Vial Number: 1
```

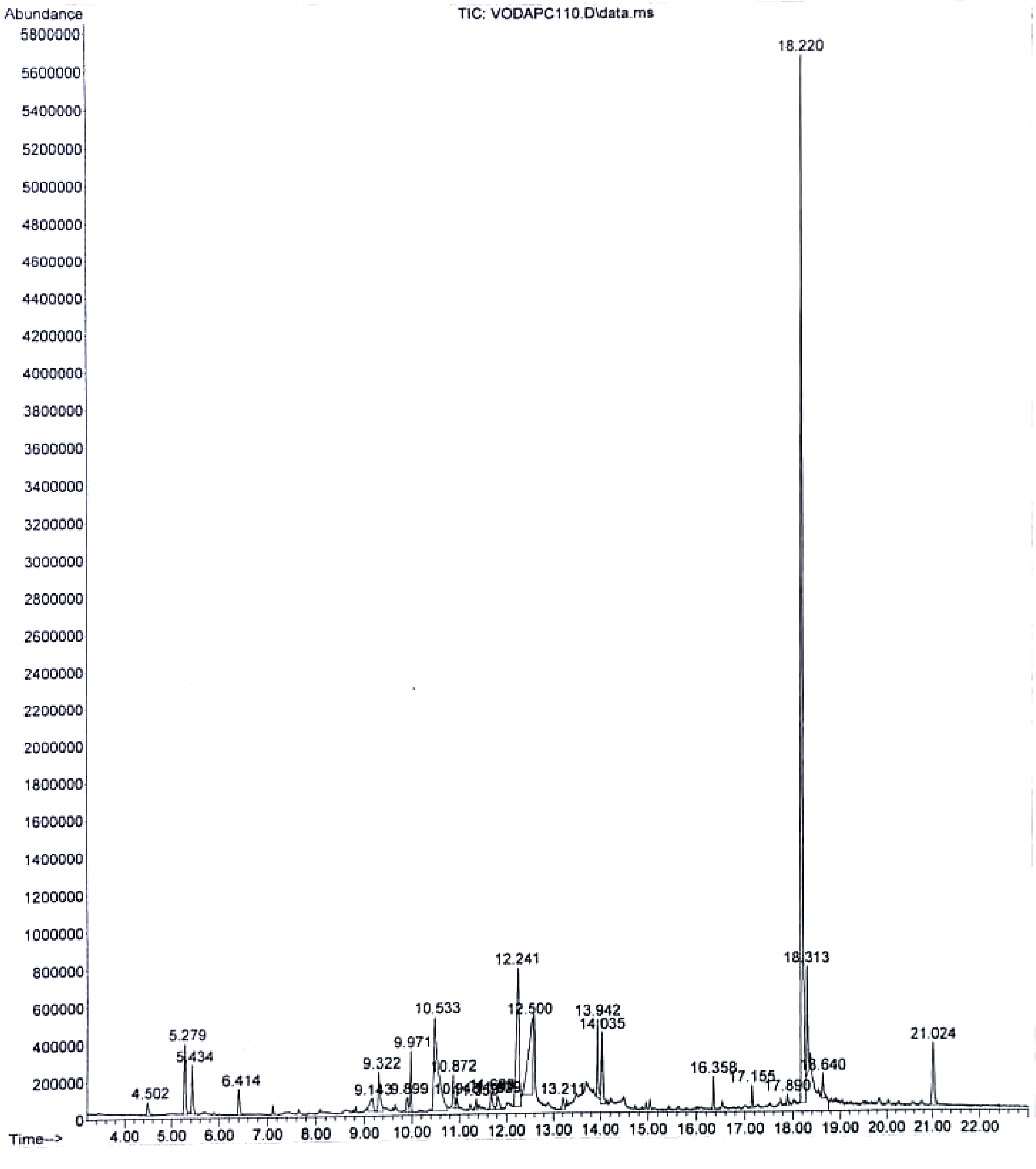

Figure S6 GC-chromatogramm of diluted Artemisia extract N4

```
File       :C:\msdchem\1\DATA\Kataliz\PCVG.D
Operator   : Lyapunov
Acquired   :  9 Jun 2022  10:30     using AcqMethod Kataliz.M
Instrument :   Instrument #1
Sample Name: PCVG    N07
Misc Info  : 1 mcl
Vial Number: 1
```

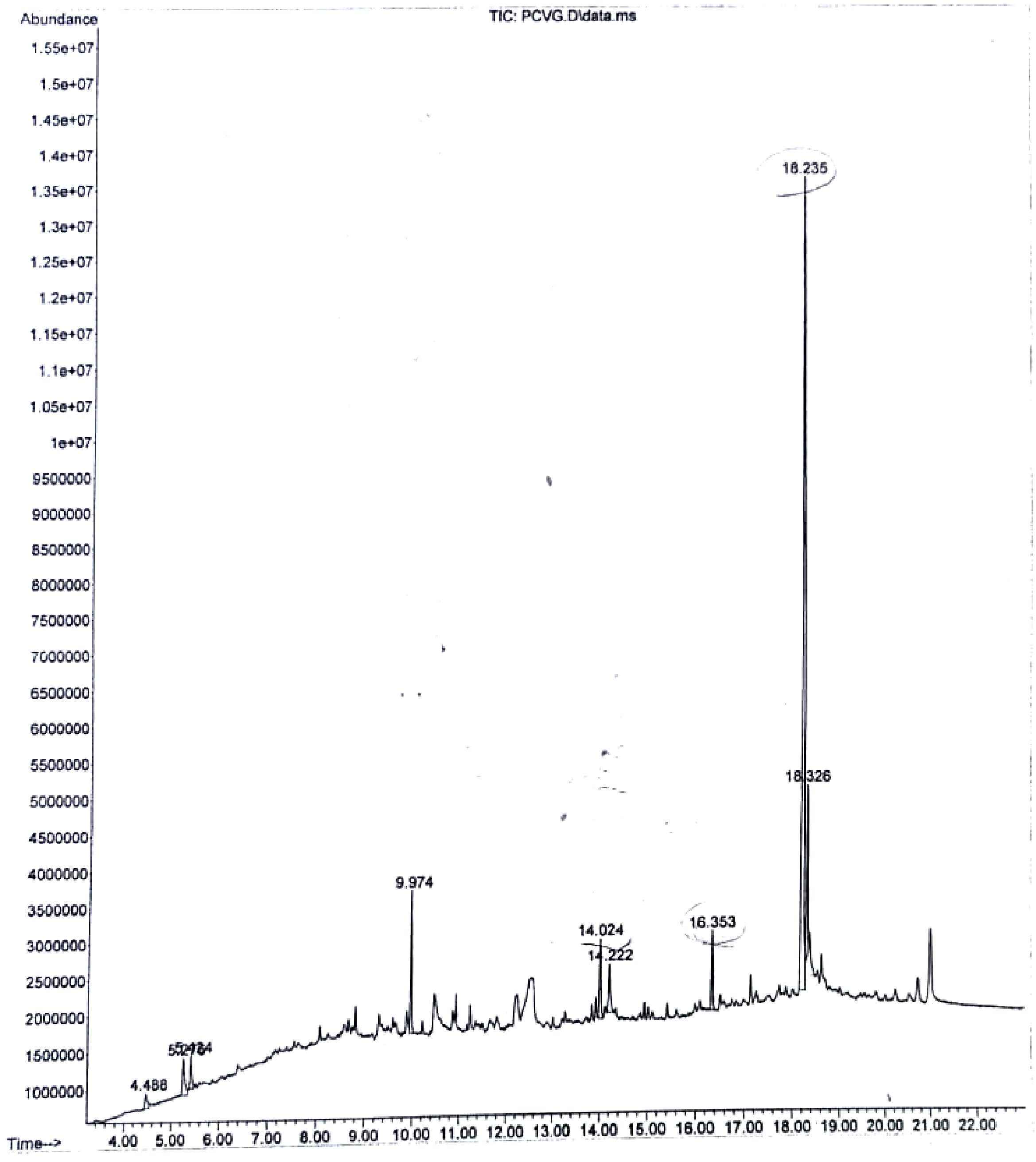

Figure S7 GC-chromatogramm of diluted Artemisia extract N7

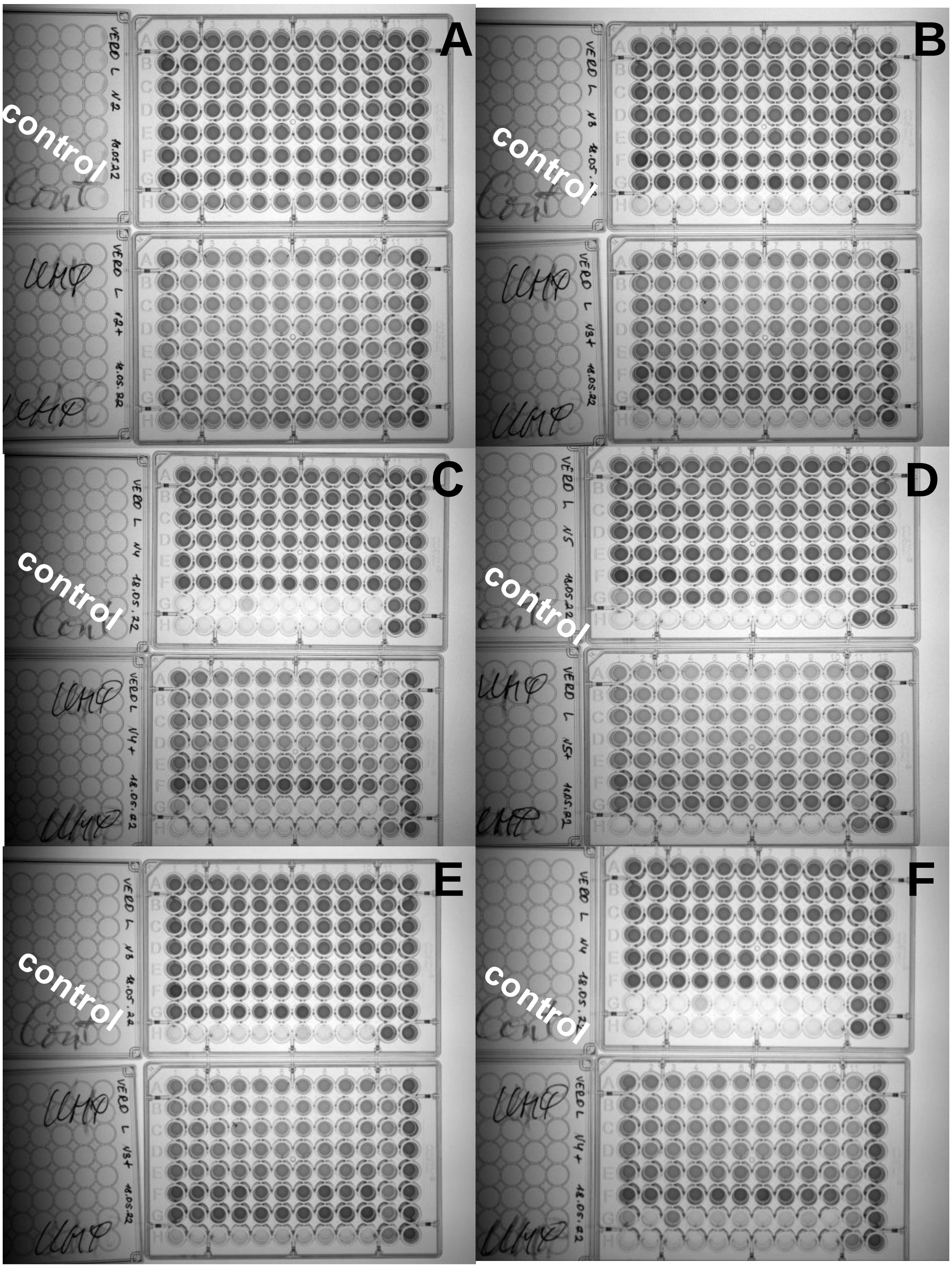

Figure S8. Photographs of 96-well plates: not infected(positive control) and SARS-CoV-2 infected sample for dilutions of Artemisia extract: A) N2; B) N3; C) N4; D) N5; E) N6; F) N7.

Table S1 – Qualitative analysis of plant raw materials of wormwood (Artemisia cina Berg) and annual wormwood

	Qualitative reactions	Expected result	Objects of research

comp ound			Wormwood boiled	Annual wormwood
Sesquiterpene lactones	By 10 mg of the resulting dry residue is dissolved in 1 ml of 96% alcohol P, 1 ml of hydroxylamine of an alkaline solution P and after 5 minutes 3-4 drops of hydrochloric acid P and 3-4 drops of an alcoholic solution of iron (III) chloride P	Formation of purple staining	Formation of intense purple staining	Formation of intense purple staining
Terpenoids	To 10 mg of the resulting dry residue , add 1 drop of a 1 % solution of vanillin P in sulfuric acid P	Formation of reddish-purple staining	Formation of red-cotton violet staining	Formation of red-cotton violet staining
Flavonoids	10 mg of the resulting dry residue is dissolved in 1 ml of 96% alcohol P, when heated with an alcoholic solution of aluminum chloride P	The upper layer, when viewed in UV light, acquires yellow-green fluorescence	When viewed in UV light, it acquires yellow-green fluorescence	When viewed in UV light, it acquires yellow-green fluorescence
Essential oils	When the sample is placed in Sudan III solution for 2-3 minutes, when viewed under a microscope, essential oils turn green.	A similar light brown spot with the corresponding Rf value should appear on the chromatogram of the test solution.	A light brown spot appeared on the chromatogram of the comparison solution, with an Rf of about 0.67.	A light brown spot appeared on the chromatogram of the comparison solution, with an Rf of about 0.67.
